# Establishment and validation of a nomogram for subsequent first-cycle live births in patients diagnosed with recurrent implantation failure: a population-based analysis

**DOI:** 10.3389/fendo.2024.1334599

**Published:** 2024-03-05

**Authors:** Yunian Zhang, Xiaoyun Gong, Manli Zhang, Yuejie Zhu, Peng Wang, Zhihui Wang, Chen Liu, Xiaolin La, Jianbing Ding

**Affiliations:** ^1^ Department of Immunology, School of Basic Medical Sciences, Xinjiang Medical University, Urumqi, China; ^2^ Center for Reproductive Medicine, First Affiliated Hospital of Xinjiang Medical University, Urumqi, China; ^3^ Xinjiang Clinical Research Centre for Reproductive Immunology, First Affiliated Hospital of Xinjiang Medical University, Urumqi, China

**Keywords:** recurrent implantation failure, *in vitro* fertilization/intracytoplasmic sperm injection transfer, nomogram model, live birth, population-based analysis

## Abstract

**Background:**

The inability of patients with recurrent implantation failure (RIF) to achieve pregnancy and a live birth after multiple high-quality embryo transfer treatments has been recognized as a major obstacle to successful application of artificial reproductive technologies. The objective of this study was to establish and validate a nomogram for prediction of subsequent first-cycle live births to guide clinical practice in patients diagnosed with RIF.

**Methods:**

A total of 538 patients who underwent *in vitro* fertilization/intracytoplasmic sperm injection treatment and were first diagnosed with RIF at the Reproductive Center of the First Affiliated Hospital of Xinjiang Medical University between January 2017 and December 2020 were enrolled. The patients were randomly divided into a training cohort (n=408) and a validation set (n=175) in a ratio of 7:3. A nomogram model was constructed using the training set based on the results of univariate and multivariate logistic regression analyses and validated in the validation set.

**Results:**

Age, body mass index, duration of RIF, endometrial thickness, type of embryo transferred, and number of previous biochemical pregnancies were included in the nomogram for prediction of subsequent first-cycle live births in patients diagnosed with RIF. Analysis of the area under the receiver-operating characteristic curve, calibration plots, and decision curve analysis showed that our predictive model for live births had excellent performance.

**Conclusion:**

We have developed and validated a novel predictive model that estimates a woman’s chances of having a live birth after a diagnosis of RIF and provides clinicians with a personalized clinical decision-making tool.

## 1 Introduction

With the rapid development of assisted reproductive technology (ART), many infertile patients can have children through *in vitro* fertilization (IVF)-embryo transfer (ET) and its expanded technologies. However, approximately 10% of women who undergo IVF experience recurrent implantation failure (RIF) (1, 2). Coughlan et al. have suggested a more complete working definition that takes maternal age, number of embryos transferred, and number of cycles completed into account (1). They define RIF as failure of clinical pregnancy after four good-quality embryo transfers with at least three fresh or frozen IVF cycles in women under the age of 40 years. The main causes of RIF include abnormal uterine anatomy, endometrial tolerance, endocrine disorders, immune factors, and chromosomal abnormalities. Given that few treatments have been shown to be effective, counseling as to the likely value of continuing with further ART represents the mainstay of current management (3, 4).

To date, more than 30 models have been developed to predict post-IVF success rates, of which Templeton’s model (5) and Nelson’s model (6) have been widely endorsed. However, the probability of a live birth after IVF-ET due to the early stage of these study and the limited reproductive technology available at the time was difficult to predict. In 2014, Luke et al. estimated the cumulative chance of a live birth per woman after three fresh treatments (7). In 2016, the McLernon model was reported to predict the likelihood of successful treatment over multiple complete cycles (8). These models all have good predictive power, but none of the current prediction models are able to predict a live birth for all women beginning their first complete cycle of IVF after diagnosis of RIF. Therefore, there is an unmet need for many couples who want to plan and prepare emotionally and financially for the next step in their treatment.

Nomograms have been widely used for medical diagnosis and evaluation of the prognosis in recent years because of their user-friendliness. In this study, we analyzed information from a large number of women under the age of 39 years with the aim of developing and validating a novel prediction model that can estimate the chances of a live birth in a woman after a diagnosis of RIF and provide an individualized clinical decision-making tool for clinicians.

## 2 Materials and methods

### 2.1 Patient selection

Information on 6,142 cycles of IVF/intracytoplasmic sperm injection (ICSI) in the Reproductive Center of the First Affiliated Hospital of Xinjiang Medical University between January 2017 and December 2020 was collected retrospectively from the electronic medical records database. For the purposes of this study, to avoid research error as far as possible, the following inclusion criteria were applied: more than three transfer fresh or frozen cycles received, at least four high-quality embryos without pregnancy, and repeating IVF (at least the fourth cycle). After excluding patients aged ≥40 years, those with uterine anomalies, autoimmune disease, such as diabetes or thyroid abnormalities, and hydrosalpinx, and couples with chromosomal abnormalities, a total of 583 cycles in 383 patients were finally included. The included patients were randomized into a training set and a validation set at a ratio of 7 to 3.

### 2.2 Controlled ovarian stimulation protocol

Protocols for induction of ovulation were tailored to each patient’s condition and ovarian reserve. The most commonly used protocols were the early follicular-phase long-acting gonadotropin hormone-releasing hormone (GnRH) agonist long protocol, the mid-luteal phase short-acting GnRH-a long protocol, antagonist protocol, and a natural cycle. We determined the initial dose of gonadotropin (Merck Serono, Bari, Italy) according to the patient’s age, body mass index (BMI), antral follicle count, and previous ovarian response to stimulation. Vaginal ultrasound is used to monitor the growth of follicles every 1–2 days, and the dosage of gonadotropins is adjusted according to the patient’s peripheral blood luteinizing hormone, estradiol, and progesterone levels to obtain as many mature oocytes as possible. When at least 1–2 dominant follicles with a diameter of ≥18 mm were observed, recombinant human chorionic gonadotropin (hCG, Livzon Pharmaceutical Group, Zhuhai, China) was injected with 4,000–10,000 U in combination with the patient’s hormone levels. Transvaginal ultrasound-guided oocyte retrieval was performed at 34–36 hours after injection of hCG. Conventional IVF or ICSI insemination was chosen depending on the male semen parameters.According to our transfer strategy, most of the fresh transfer cycles were D3 and a few were D5 and D6 blastocysts embryos,while frozen ET cycles included D3, D5 and D6 embryos.

### 2.3 Preparation protocols for frozen-thawed ET

Transvaginal ultrasound and basic endocrinological examination was performed on day 2 to 3 of the menstrual cycle to monitor follicular development and endometrial condition starting. If there is no obvious abnormality, start to take estradiol (4-6mg/d) orally and adjust the medication according to the patient’s endometrial condition. The dose of estradiol was maintained when the endometrial thickness was ≥8 mm, and the duration of administration was ≥12 days, while luteal support was given at the same time. Endometrial transformation day was counted as day 0 (D0), on day 3 (D3) or day 5 (D5) and day 6 (D6) for the transfer of cleavage-stage or blastocyst-stage embryos, respectively.

### 2.4 Embryo scoring

Normal fertilization was confirmed when the presence of two pronuclei and the extrusion of the second polar body were observed 16–18 h after IVF/ICSI (9, 10).

According to the Peter score assessment, cleavage embryos were classified as high-quality (grade I or II) if they had 7–9 cells on day 3 and as same-sized blastomeres if they had less than 20% blastomeric fragments. Embryos graded III or IV, including those that had fewer than 7 cells on day 3, and those without less than 20% fragmentation were considered to be of poor quality (11).

Blastocysts were evaluated using the Gardner grading system (12). Blastocysts were classified into stages 1–6 according to their development and stage status (1, blastocele cavity less than half the volume of the embryo; 2, blastocele cavity more than half the volume of the embryo; 3, full blastocyst, cavity completely filling the embryo; 4, expanded blastocyst, cavity larger than the embryo with thinning of the shell; 5, hatching out of the shell; 6, hatched out of the shell). When the blastocyst reached stage ≥3, the inner cell mass was rated (A, many cells, tightly packed; B, several cells, loosely grouped; C, very few cells) and the trophectoderm was rated (A, many cells, forming a cohesive layer; B, few cells, forming a loose epithelium; C, very few large cells). At our center, blastocysts that develop to stage 3–6 on day 5–6 with an inner cell mass quality grading ≥B are considered as freezable blastocysts and those with a score ≥3BB on day 5–6 are designated as high-quality blastocysts.

### 2.5 Luteal phase support regimen

Luteal support was provided by daily administration of vaginal progesterone gel (Crinone, Merck & Serono) at a dosage of 90 mg, along with a once-daily oral dose of 10 mg dydrogesterone(Duphaston, AbbottBiologicalsB.V) which was gradually tapered off after pregnancy and discontinued by the 12th week of gestation.

### 2.6 Baseline characteristics

Couple demographics and clinical characteristics at the start of the transplant cycle after diagnosis of RIF were used as baseline parameters, included age (years), BMI, duration of RIF (months), type of infertility (primary or secondary), cause of infertility (tubal, uterine, ovulatory, male, or unexplained), type of treatment (IVF, ICSI, or half-ICSI), endometrial thickness (mm, measured in the midsagittal plane of the uterine body on the day of administration of hCG), ovarian hyperstimulation protocol (long, short, antagonist, or other), type of embryo transferred (cleavage embryo or blastocyst), and numbers of previous biochemical pregnancies and intrauterine procedures.

The date of diagnosis of RIF is defined as the first day of menstruation after having received more than three fresh or frozen transfer cycles and at least four high-quality embryos without pregnancy.

### 2.7 Primary outcome and sample size considerations

The primary outcome of the nomogram was live birth, defined as a pregnancy that continues with at least one live-born fetus after 28 weeks of gestation and survival for more than one month. In order to fit the predictive model using logistic regression, a minimum of 10 events (ie,subsequent first-cycle live births after RIF) per variable (EPV) are recommended (13–15). We evaluated 12 variables in the logistic regression model, so the sample size in the derivation stage was at least 120 events. Based on the results of the study by Yuan Fang et al., we assumed a live birth rate of 25% after a diagnosis of RIF (16). Therefore, we planed to collect at least 480 cases.

### 2.8 Statistical analysis

All statistical analyses were performed using R software (version 4.0.2; The R Foundation for Statistical Computing, Vienna, Austria) and Excel (Microsoft Corp., Redmond, WA, USA). The patients were divided into a training set and a validation set by the random numbers sampling technique. Continuous variables are expressed as the mean ± standard deviation or the median (interquartile range) and were compared using the *t*-test, analysis of variance, or Mann–Whitney *U* test. Categorical variables are presented as the proportion and were compared using the chi-squared test or Fisher’s exact test to identify candidate covariates. All potential confounders with a two-sided P-value <0.1 were then entered into a multivariate logistic regression model based on the forward stepwise method to establish the probability of a live birth in the first cycle after diagnosis of RIF. Variables with a P-value <0.05 were considered to be independent predictive factors and used to develop a dynamic nomogram using the “Dynnom” package.

The nomogram scoring system was evaluated as follows: discriminatory power and accuracy using the area under the receiver-operating characteristic (ROC) curve; prediction accuracy according to the calibration curve; and potential clinical applications by decision curve analysis.

## 3 Results

### 3.1 Analysis of patients’ baseline characteristics

**Figure 1:**
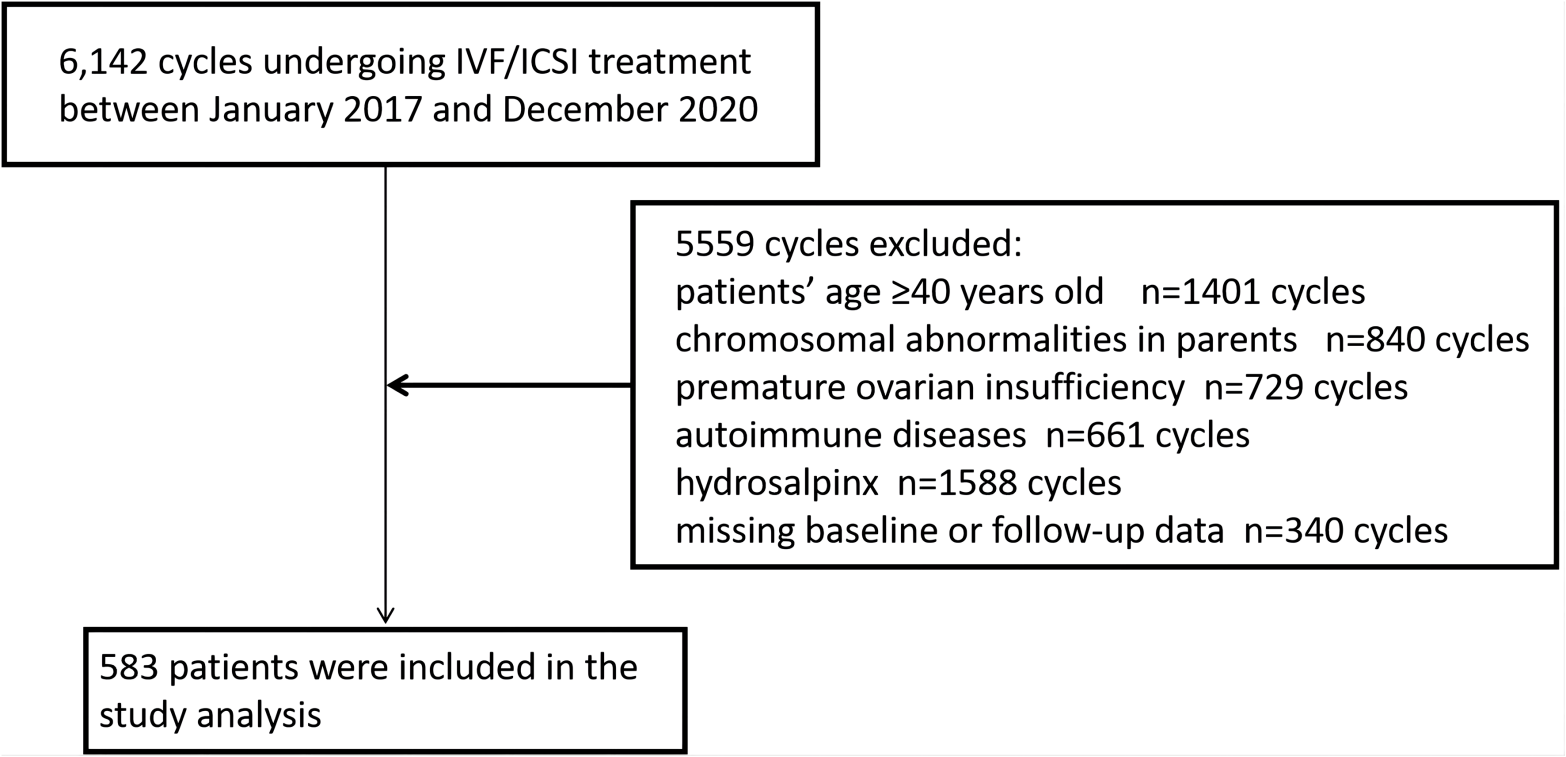
Flowchart showing the patient enrolment process. ICSI, intracytoplasmic sperm injection; IVF, *in vitro* fertilization.

Among the 6,142 IVF/ICSI cycles performed between January 2017 and December 2020, 5559 cycles met one of the following exclusion criteria so were not available for analysis: age ≥40 years (n=1401), chromosomal abnormalities in the parents (n=840); premature ovarian insufficiency (n=729), autoimmune disease (n=661), hydrosalpinx (n=1588), and missing baseline or follow-up data (n=340). Finally, RIF was identified in 583 cycles (**Figure 1**). A total of 408 cycles (70%) were assigned to the training set and 175 (30%) to the validation set. The baseline information for patients with RIF in the training and validation sets are shown in **Table 1**. There were no statistically significant differences between the two groups (P>0.05).

**Table 1:** Baseline characteristics in the total set.

Characteristics	Training set (n=408)	validation set (n=175)	P-value
Age(year)	32.78 ± 4.22	32.56 ± 4.02	0.559
BMI(kg/m2)	22.51 ± 3.01	22.30 ± 2.81	0.427
Duration of RIF(month)	5.11 ± 1.24	5.25 ± 1.37	0.233
Type of infertility			0.315
Primary infertility	159(39.0%)	76(43.4%)	
Secondary infertility	249(61.0%)	99(56.6%)	
Cause of infertility			0.866
Tubal factor	45(11.0%)	18(10.3%)	
Uterine factors	66(16.2%)	24(13.7%)	
Ovulatory factor	65(15.9%)	31(17.7%)	
Male factor	53(13.0)	20(11.4%)	
Combined factor	89(21.8%)	45(25.7%)	
Unexplained	90(22.1%)	37(21.3%)	
Type of treatment			0.893
IVF	153(37.5%)	62(35.4%)	
ICSI	178(43.6%)	79(45.1%)	
IVF+ICSI	77(18.9%)	34(19.4%)	
Emdometrial thickness(mm)			0.279
<8	34(8.3%)	19(10.9%)	
8-13	283(69.4%)	126(72.0%)	
>13	91(22.3%)	30(17.1%)	
Ovarian hyperstimulation/Endometrial preparation protocols			0.057
Long protocol	205(50.2.6%)	102(58.3%)	
Short protocol	64(15.7%)	23(13.1%)	
Antagonist protocol	67(16.4%)	33(18.9%)	
Other protocol	72(17.6%)	17(9.7%)	
Type of embryo transferred			0.377
Cleavage embryo	250(61.3%)	158(38.7%)	
Blastocyst	114(61.3%)	61(34.9%)	
Number of oocytes retrieved	12.70 ± 4.94	12.56 ± 5.39	0.763
Number of 2PN	8.97 ± 5.12	9.05 ± 5.37	0.864
Number of embryos transferred			0.172
1	162(39.7%)	59(33.7%)	
2	246(60.3%)	116(66.3%)	
Previous biochemical pregnancy			0.848
0	305(74.8%)	131(74.9%)	
1	70(17.2%)	32(18.3%)	
≥2	33(8.0%)	12(6.9%)	
Number of intrauterine procedures	1.23 ± 0.528	1.19 ± 0.507	0.434

2PN, two pronuclei; BMI, body mass index; ICSI, intracytoplasmic sperm injection; IVF, in vitro fertilization; RIF, recurrent implantation failure.

### 3.2 Logistic regression analysis of live births

**Figure 2:**
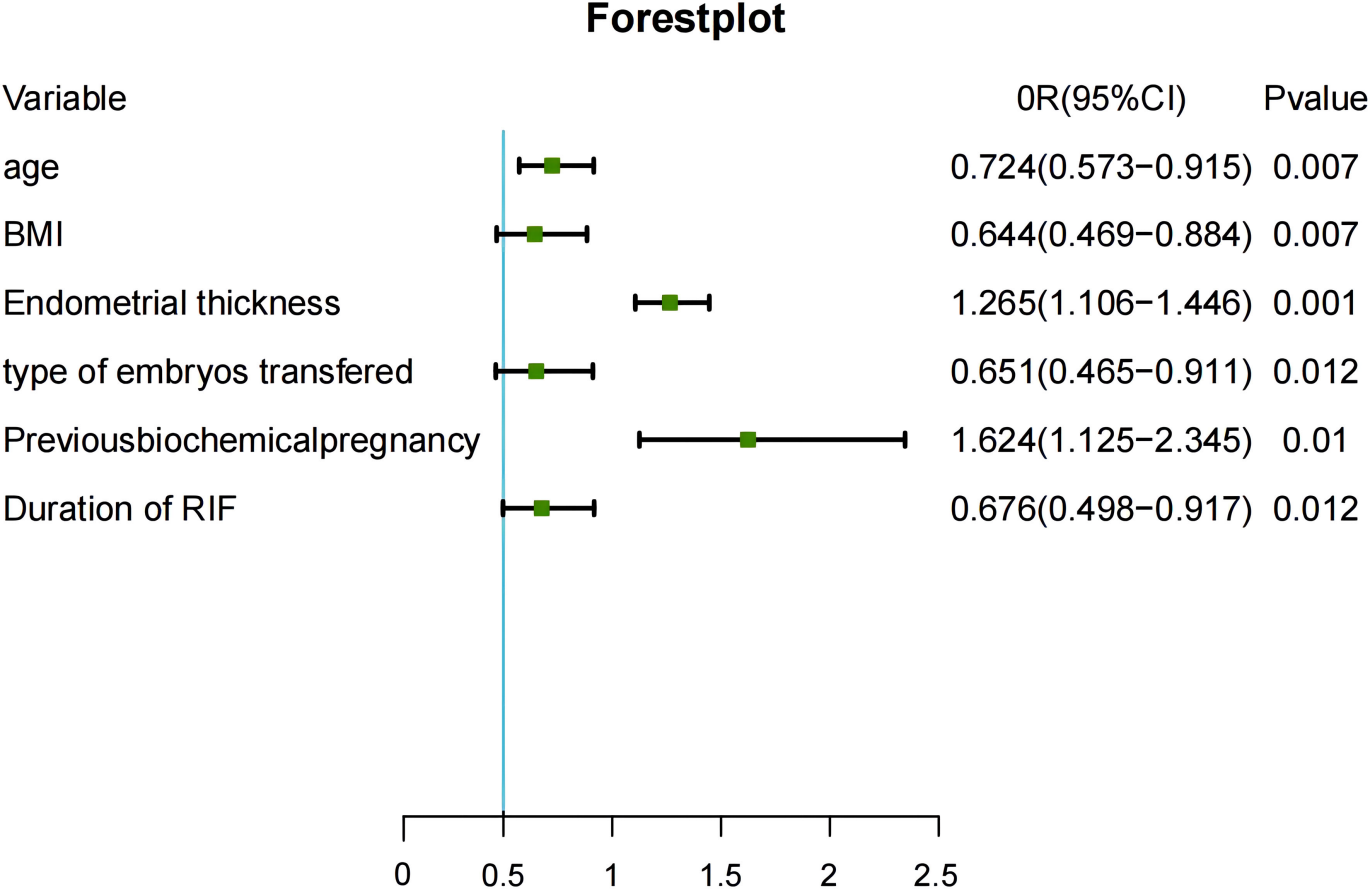
Risk factors identified in multivariable logistic regression analysis. CI, confidence interval; BMI, body mass index; OR, odds ratio; RIF, recurrent implantation failure.

**Table 2** compares the baseline characteristics between the live birth group and the no live birth group in the training set. According to the univariate analysis, live births in patients with RIF were associated with age, BMI, duration of RIF, endometrial thickness, type of embryo transferred, and number of previous biochemical pregnancies (P<0.05). Statistically significant variables identified in the univariate analysis were entered into the unconditional binary multivariate logistic regression model. Multivariate logistic regression analysis identified female age (odds ratio [OR] 0.724, 95% confidence interval [CI] 0.573–0.915), BMI (OR 0.644, 95% CI 0.469–0.884), duration of RIF (OR 0.676, 95% CI 0.498–0.917), endometrial thickness (OR 1.265, 95% CI 1.106–1.446), type of embryo transferred (OR 0.651, 95% CI 0.465–0.911), and previous biochemical pregnancy (OR 1.624, 95% CI 1.125–2.345) to be significantly associated with a live birth in patients with RIF. The results of the multivariable logistic regression analysis are shown as forest plots in **Figure 2**.

**Table 2:** Baseline characteristics in the training set.

	Live birth (n=157)	No live birth (n=251)	P-value
Age(year)	32.08 ± 3.93	33.19 ± 3.43	<0.001
BMI(kg/m2)	21.88 ± 2.95	22.58 ± 2.99	0.032
Duration of RIF(month)	4.90 ± 1.31	5.46 ± 1.45	<0.001
Type of infertility			0.64
Primary infertility	61(38.9%)	103(41.0%)	
Secondary infertility	96(61.1%)	148(59.0%)	
Cause of infertility			0.733
Tubal factor	17(10.8%)	27(10.8%)	
Uterine factors	23(14.6%)	40(15.9%)	
Ovulatory factor	22(14.0%)	46(18.3%)	
Male factor	20(12.7%)	31(12.3%)	
Combined factor	38(24.2%)	56(22.3%)	
Unexplained	37(23.6%)	51(20.3%)	
Type of treatment			0.764
IVF	70(44.6%)	107(42.6%)	
ICSI	56(35.7%)	98(39.0)	
Half-ICSI	31(19.7%)	46(18.3%)	
Endometrial thickness(mm)			<0.001
<8	10(6.4%)	27(10.8%)	
8-13	128(80.9%)	158(62.9%)	
>13	19(12.1%)	66(26.3%)	
Ovarian hyperstimulation protocols			0.946
Long protocol	84(53.5%)	131(52.2%)	
Short protocol	24(15.3%)	37(14.7%)	
Antagonist protocol	27(17.2%)	43(17.1%)	
Other protocol	22(14.0%)	40(15.9%)	
Type of embryo transferred			0.028
Cleavage embryo	89(56.7%)	164(65.3%)	
Blastocyst	68(43.3%)	87(34.7%)	
Number of embryos transferred			0.089
1	66(42.0%)	88(35.1%)	
2	91(58.0%)	163(64.9%)	
Previous biochemical pregnancy			<0.001
0	101(64.3%)	178(70.9%)	
1	46(29.3%)	42(16.7%)	
≥2	10(6.4%)	31(12.4%)	
Number of intrauterine procedures	1.14 ± 0.447	1.28 ± 0.55	0.853

BMI, body mass index; ICSI, intracytoplasmic sperm injection; IVF, in vitro fertilization; RIF, recurrent implantation failure.

### 3.3 Development and validation of the nomogram

**Figure 3:**
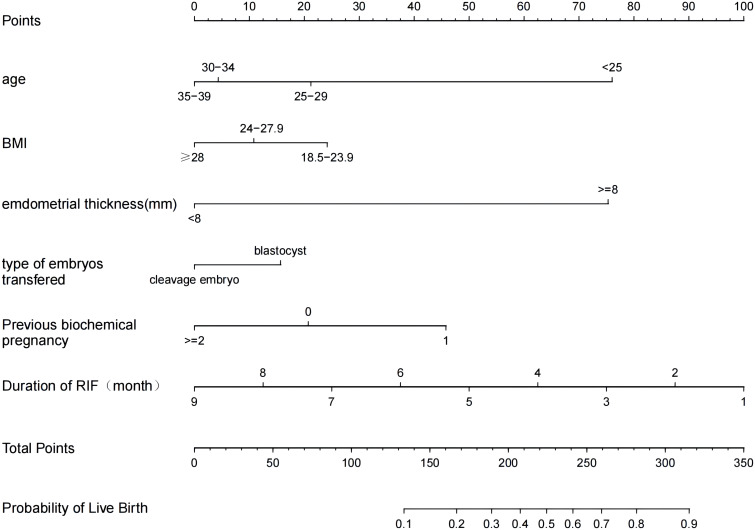
Nomogram for predicting the probability of a live birth in patients with RIF. BMI, body mass index; RIF, recurrent implantation failure.

We included six independent factors in the prediction model. Next, the training set was used to build the individualized nomogram prediction model. According to the nomogram, the corresponding score for each predictor was obtained. The probability of a live birth in patients with RIF could be derived from the sum of these scores, as shown in **Figure 3**.

**Figure 4:**
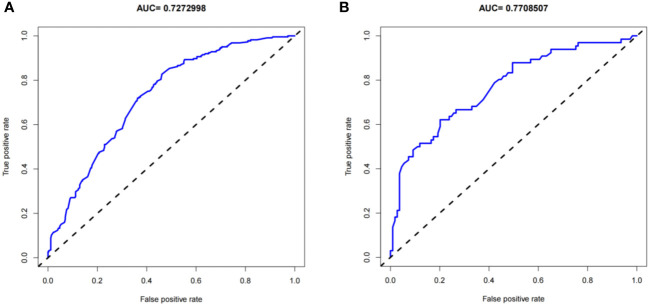
Receiver-operating characteristic curves for the live birth prediction model in patients with recurrent implantation failure. **(A)** training set and **(B)** validation set. AUC, area under the curve.

**Figure 5:**
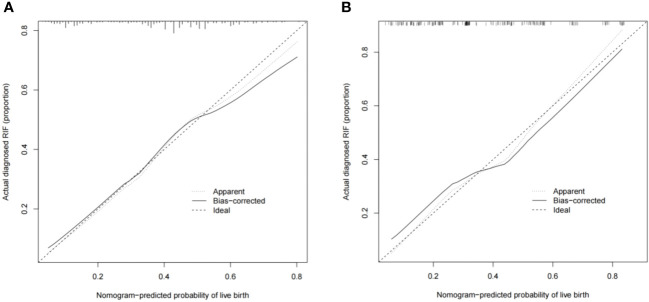
Calibration plots showing the nomogram-predicted probability of a live birth in the **(A)** training set and **(B)** validation set.

**Figure 6:**
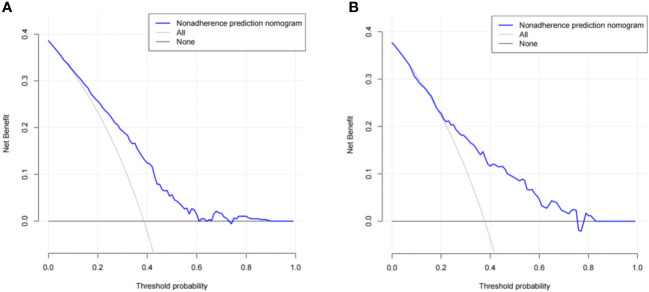
Results of decision curve analysis using the nomogram in the **(A)** training set and **(B)** validation set.

The predictive effect of the nomogram scoring system was evaluated using the area under the ROC curve and area under the curve (AUC). The area under the ROC curve for the live birth prediction model for patients with RIF was 0.727 in the training set and 0.771 in the validation set (**Figure 4**). The AUC indicated that the nomogram model is helpful for discrimination. The calibration curve illustrated an overlap between the probabilities of predicted diagnosis and actual diagnosis of stroke in both the training set and the validation set (**Figure 5**). The decision curves showed that the quantified the net benefits of the training and validation sets was higher when the probability was between 50% and 70%. Furthermore, the results of the decision curve analysis indicated that the nomogram model had good clinical utility (**Figure 6**).

## 4 Discussion

Patients with RIF are a special group in the infertile population who have undergone multiple high-quality embryo transfer treatments but still cannot achieve pregnancy and a live birth. It is now believed that development of a personalized IVF regimen may be the most effective way of improving pregnancy outcomes in these patients. Over the past three decades, several predictive models have been applied to contemporary clinical and laboratory IVF protocols, but it is still not possible to predict subsequent cycles in patients with RIF. Therefore, we have developed a highly discriminative and practical predictive model that uses existing clinical data to predict the chances of a subsequent first-cycle live birth in a patient diagnosed with RIF. The nomogram identified the predictors in our model to be female age, BMI, duration of RIF, endometrial thickness, type of embryo transferred, and previous biochemical pregnancy.

Multiple studies have consistently shown that maternal age is a significant independent prognostic factor in terms of the outcome of IVF (1). Advanced female age not only leads to a decrease in the number of transferable embryos but also has a profound effect on the quality of embryos, especially the increase in the number of aneuploid embryos, which significantly reduces the pregnancy rate (17, 18). Salumets et al. found that maternal age was a major factor affecting pregnancy outcome in frozen ET, especially with the ICSI technique, and that the patient’s age at the time of oocyte collection and freezing was the only determinant of biochemical pregnancy outcome (19). In women under the age of 35 years, the likelihood of a biochemical pregnancy between 6 and 12 weeks of gestation ranged from 9% to 12%. This risk escalated in women over the age of 35 years, largely because of a significantly higher prevalence of trisomic pregnancies. Remarkably, for women over 40 years of age, the incidence of trisomy has been reported to reach nearly 50%, further exacerbating the risk (20). Furthermore, Shapiro and Daneshmand found that the rates of embryo-endometrial asynchrony also increase with increasing maternal age (21). In their study, transfers were asynchronous in 50% of women under 35 years of age and in 68.1% of those over this age Their findings lend support to the notion that embryo quality, including genetic characteristics, declines with increasing maternal age. By using the nomogram developed in this study, we can predict that the subsequent first-cycle live birth rate in women with a diagnosis of RIF decreases with advancing age.

Our study found that higher BMI levels were associated with a reduced likelihood of live births in patients with RIF. This finding is consistent with previous research suggesting that patients with a high BMI often have metabolic dysfunction, which affects the function of organs and tissues and damages the patient’s vascular endothelium (22), which triggers a chain reaction. First, it induces a state of systemic inflammation throughout the body, which in turn activates the immune system, making it more difficult for an embryo to implant successfully in the uterus. Simultaneously, the compromised vascular endothelium activates coagulation factors that interfere with the blood supply. This process is critical because a well-functioning endometrial lining is essential for nourishing the embryo and facilitating its implantation. Our findings support the view that elevated BMI is the most important risk factor after increasing maternal age contributing to miscarriage among patients with RIF (23, 24).

A prolonged duration of RIF is another important risk factor in patients undergoing ART-assisted fertility treatment. It has been shown that duration of RIF has a significant negative correlation with the outcome of pregnancy (25). As the duration of RIF increases, the patient’s stress level may also increase further, leading to a higher incidence of adverse pregnancy outcomes. Our present findings are consistent with another report (26) indicating that continuation of ART-assisted fertility treatment as soon as possible after a diagnosis of RIF leads to a significant reduction in the interval between pregnancies achieved in patients with RIF.

Endometrial thickness can reflect the functional status of the endometrium during IVF-ET. A meta-analysis of 22 clinical studies (10,724 IVF-ET cycles) found that the embryo implantation and clinical pregnancy rates were lower in patients with a thin endometrium (<7 mm) on the day of hCG or the day of implantation than in patients with a thicker endometrium (>7 mm) (27). Some study found that patients with an endometrial thickness of 8.7–14.5 mm who underwent hormone replacement therapy and thawed ET had the best live birth outcomes (28). Our results also included a significant increase in the live birth rate in patients with an endometrial thickness ≥8 mm on the day of embryo transfer in comparison with patients with an endometrial thickness of <8 mm.

Unexplained RIF is usually attributable to abnormalities in gametes (sperm and oocyte) and the embryo. An association has been found between RIF and DNA damage in sperm, which can lead to failure of blastocyst hatching if severe (29, 30). Oocytes provide half of the genetic material and all the energy supply for the embryo, and the number and function of their mitochondria and the functional status of their surrounding granulosa cells are closely related to blastocyst formation and embryo development. Several reports has shown that blastocyst transfer, by prolonging *in vitro* culture of embryos, can further screen embryos while keeping the developmental stage of the embryo relatively synchronized with the endometrium, which in turn can improve the rate of embryo implantation (31, 32). The results of our study confirm that an extended *in vitro* culture time for blastocyst culture in patients with unexplained RIF, who may have embryonic defects owing to sperm, oocytes or embryonic factors, facilitates further embryo screening and thus improves the outcome of assisted conception.

Previous studies have suggested that a history of biochemical pregnancy in IVF cycles predicts poor pregnancy outcomes in subsequent ART cycles, including significantly higher rates of biochemical pregnancy and miscarriage (33, 34). However, in our study, multivariate logistic regression analyses showed that patients with a history of a biochemical pregnancy may have better conception outcomes in subsequent RIF cycles. Of note, we found that the live birth rate was significantly lower when there were two or more biochemical pregnancies. This result is similar to that in a study by Haas and Lerner-Geva in patients with repeated IVF failure. They found that biochemical pregnancies in the first three cycles did not significantly alter the chances of pregnancy (35). However, from the fourth cycle onwards, a previous biochemical pregnancy significantly increased the rate of sustained pregnancy.

The predictive model developed in this study is highly discriminative, well-calibrated, and practical, and will allow clinicians to use existing clinical data to predict subsequent first-cycle live births in patients diagnosed with RIF. However, this study had a number of limitations. First, it was performed at a single center, which may limit the generalizability of the findings. Second, it had a retrospective design, which may have introduced a degree of data bias. Although patient samples from different time periods were used to validate the model, there is still a need for evidence from other centers for validation. Therefore, we will perform a prospective multicenter study for further in-depth evaluation and validation of this prediction model.

In conclusion, we have developed and validated an objective and accurate nomogram that can be used to predict subsequent first-cycle live births in patients diagnosed with RIF. Clinicians can perform a relative risk assessment during infertility consultations and take appropriate measures in advance to minimize the likelihood of fetal loss.

## Data availability statement

The original contributions presented in the study are included in the article/supplementary material. Further inquiries can be directed to the corresponding authors.

## Ethics statement

The studies involving humans were approved by Ethics Committee of the First Affiliated Hospital of Xinjiang Medical University. The studies were conducted in accordance with the local legislation and institutional requirements. The participants provided their written informed consent to participate in this study.

## Author contributions

YnZ: Writing – original draft. XG: Writing – original draft. MZ: Data curation, Writing – review & editing. YuZ: Data curation, Writing – review & editing. PW: Formal analysis, Writing – review & editing. ZW: Formal analysis, Writing – review & editing. CL: Visualization, Writing – review & editing. XL: Writing – review & editing, Funding acquisition. JD: Writing – review & editing.
